# Rheological properties of composite polymers and hybrid nanocomposites

**DOI:** 10.1016/j.heliyon.2020.e04187

**Published:** 2020-06-13

**Authors:** Rachid Hsissou, Atiqa Bekhta, Omar Dagdag, Abderrahim El Bachiri, Mohamed Rafik, Ahmed Elharfi

**Affiliations:** a Laboratory of Advanced Materials and Process Engineering, Faculty of Sciences, Ibn Tofail University, BP 242, 14000, Kenitra, Morocco; b Laboratory of Industrial Technologies and Services, Height School of Technology, Sidi Mohammed Ben Abdallah University, Fez, Morocco; c Royal Naval School, University Department - Boulevard Sour- Jdid, Casablanca, Morocco

**Keywords:** Chemical engineering, Nanotechnology, Organic chemistry, Materials chemistry, Polymer, Nanocomposite, Fiber, Nanoparticles, Viscosimetric, Viscoelastic, Rheological

## Abstract

This paper summarizes a review of the viscosimetric, viscoelastic and rheological properties of polymers and hybrid nanocomposite polymers. Hybrid nanocomposites can be combined from natural fibers or synthetic fibers and/or both. The hybrid nanocomposite polymer offers the designer the opportunity to achieve the required characteristics to a considerable extent controlled by the choice of appropriate fibers or fillers and the polymer architecture. The rheological behavior of hybrid nanocomposite depends on fiber content, fiber length, fiber orientation, fiber-to-matrix bonding, fiber configuration and filler, respectively. Further, rheological properties of hybrid nanocomposite polymers by introducing various charges were examined discussed.

## 1 Introduction

Hybrid nanocomposite polymers are more developed than traditional fiber-reinforced nanocomposite polymers [1, 2]. These reinforced nanocomposites contain a single reinforcement phase in the single polymeric matrix however hybrid composites may have more than one reinforcement phase and a single matrix phase or a single reinforcement phase with multiple matrix phases [3, 4]. Further, hybrid nanocomposites can have fillers of natural or synthetic origin. Natural fibers have superior rheological properties such as rigidity, flexibility etc... Their main advantages are low cost, light weight, easy and environmentally friendly production [5, 6]. However, the addition of nanoscale synthetic fibers in nanoparticles form can improve the mechanical properties of the polymer such as rigidity and compressive strength. Also, it can be improving the viscosimetric, viscoelastic and rheological properties of polymer and its nanocomposites [7]. Hybrid nanocomposite materials can produce high rheological properties such as high specific strength, stiffness and very good workability [8, 9]. High performance nanocomposites have various applications such as aircraft processing, wind turbine blades, automotive, intelligent memory, ship structures and bridge construction [10, 11, 12]. Polymeric nanocomposites reinforcing by various loads such as silica covered by argent nanowires, silica covered by carbon nanotube, multi-walled carbon nanotube, silicone rubber and boron nitride nanosheets presented an excellent rheological and thermal properties [13, 14, 15, 16, 17]. The objective of this present work is to conduct a careful review on viscosimetric, viscoelastic and rheological behaviors of polymers and hybrid nanocomposite polymers.

## 2 Viscosimetric properties

### 2.1 Viscosity of polymers only

**Figure 1:**
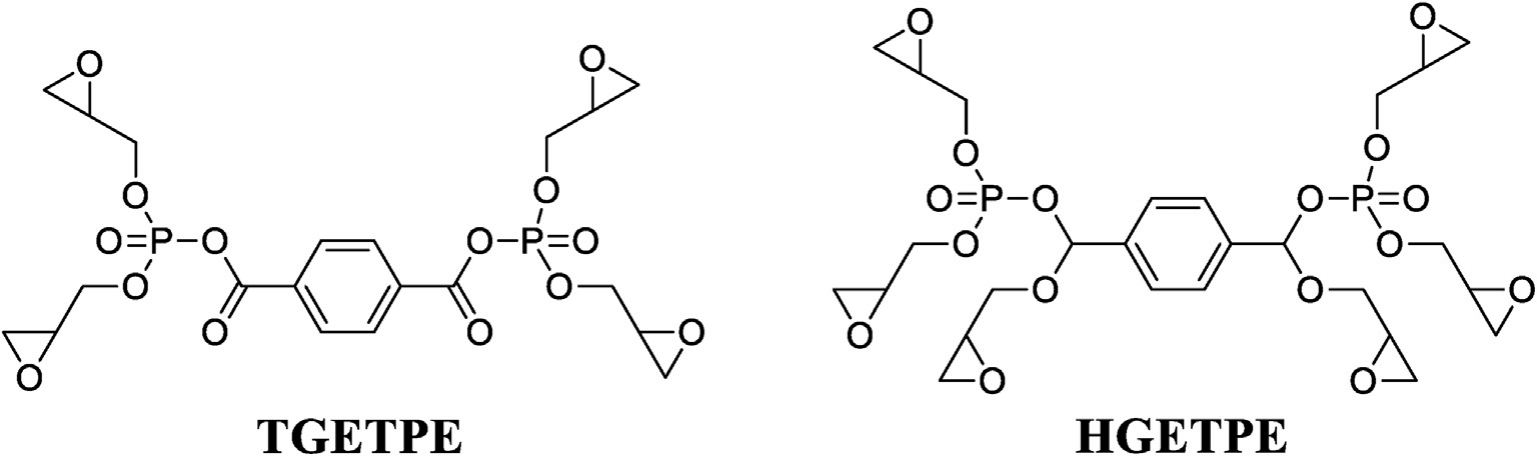
Structures of TGETPE and HGETPE [18].

Ziraoui et al. [18] studied the viscosimetric behavior at different temperatures of tetra and hexafunctional epoxy polymers such as tetraglycidyl ether terephthalydene bis-para-phosphoric ester (TGETPE) and hexaglycidyl ether terephthalydene bis-para phosphoric ester (HGETPE) (Figure 1) [18]. TGETPE and HGETPE were synthesized and identified by Ziraoui et al. [18]**.** These viscosity behaviors could be connected to the macromolecular structure of the reacting system. These two epoxy polymers diluted in chloroform have almost the same viscosimetric behavior whatever the temperature of the systems (polymer/solvent). They showed that the viscosimetric behavior elevates by elevating the mass percentage of the polymer studied, due to the progression of the homopolymerization reaction, while confirming that storage of tetra and hexafunctional epoxy polymers takes place at temperatures above 45 °C.

### 2.2 Viscosity of (polymer/solvent) systems

**Figure 2:**
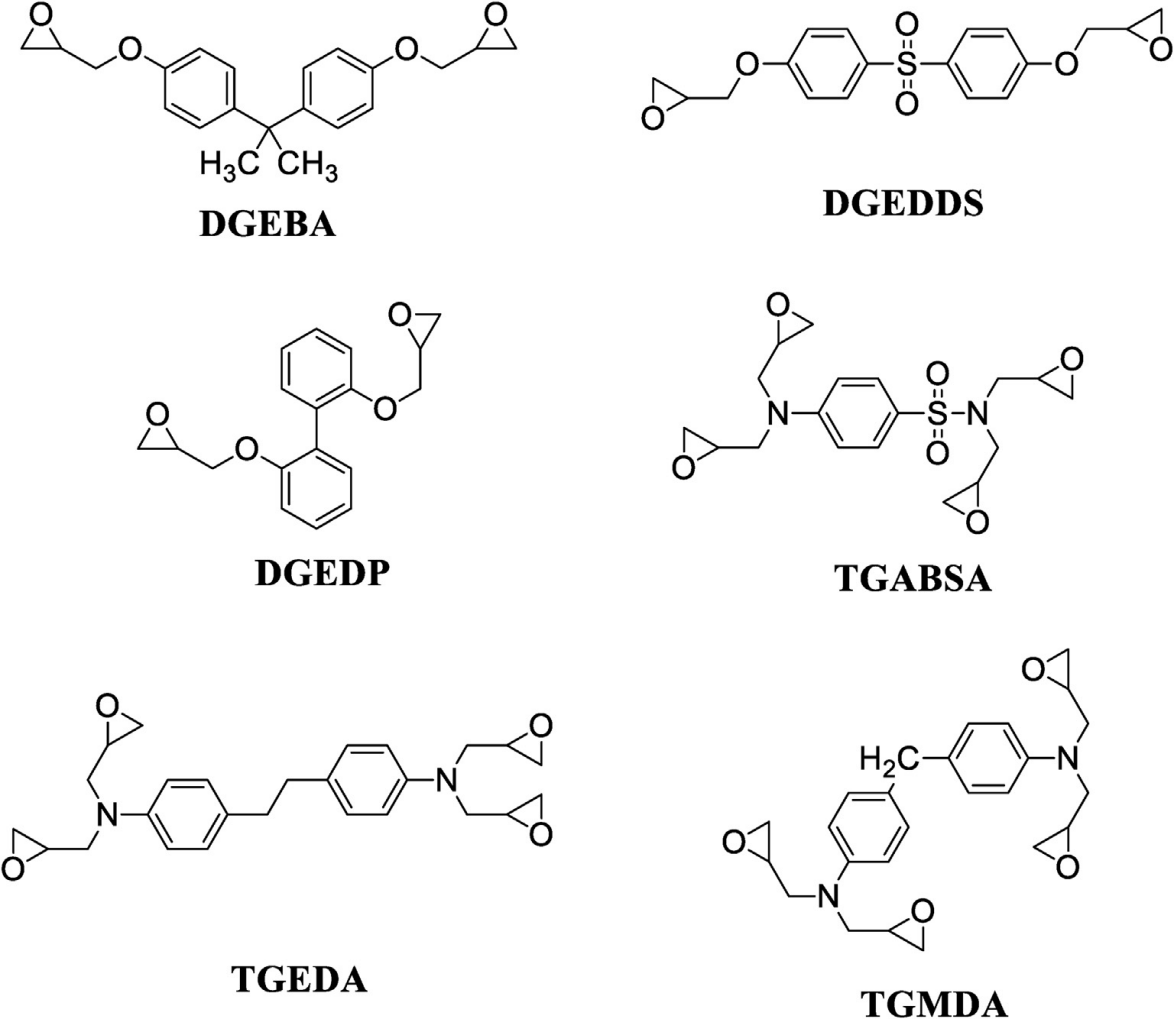
Structures of DGEBA, DGEDDS, DGEDP, TGABSA, TGEDA and TGMDA [19].

**Figure 3:**
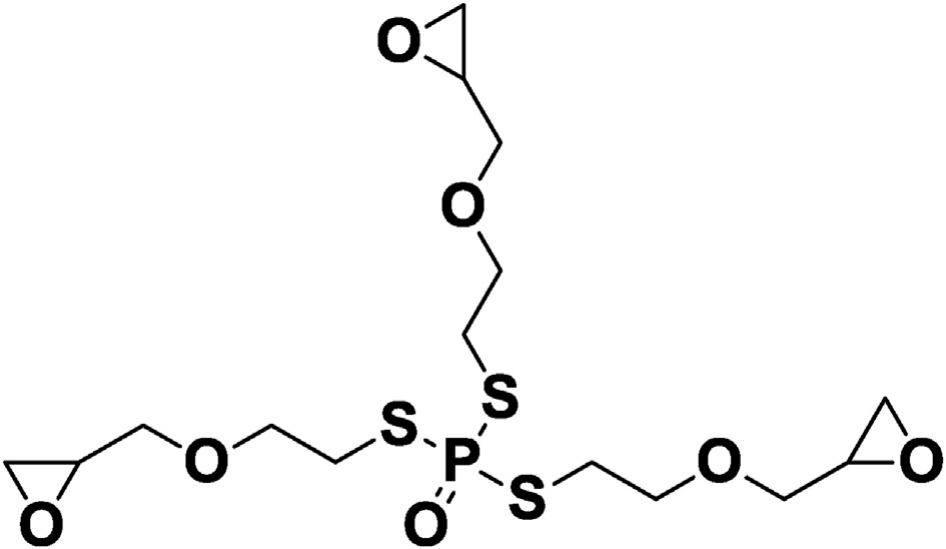
Structure of TGETMEP [20].

**Figure 4:**
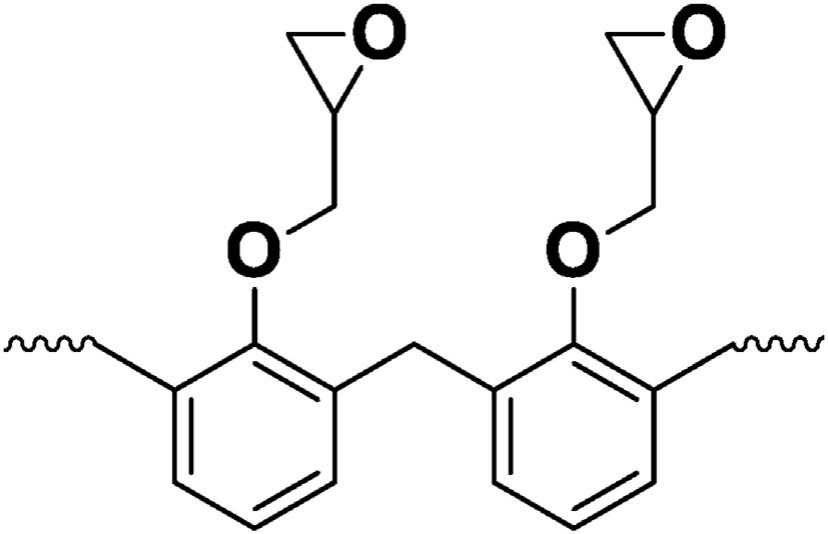
Structure of NEP [23, 24].

**Figure 5:**
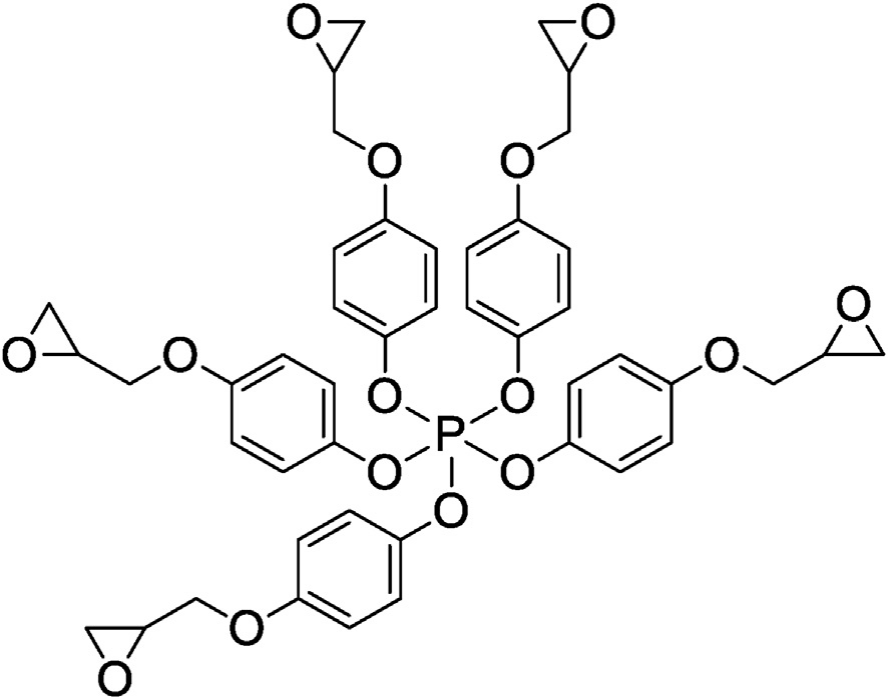
Structure of PGEPPP [25].

El Gouri et al. [19] studied the viscosimetric behavior as a function of temperature of different epoxy prepolymers such as diglycidyl ether of bisphenol A (DGEBA), diglycidyl ether of diphenylsulfone (DGEDDS), diglycidyl ether of diphenyl (DGEDP), tetraglycidyl methylene dianiline (TGMDA), tetraglycidyl ethylene dianiline (TGEDA) and tetraglycidyl 4-amino benzene sulfonamide (TGABSA) (Figure 2) [19]. These epoxy polymers were developed and characterized by El Gouri et al. [19]**.** They showed that the rheological properties of phenolic polymers dissolved in chloroform (DGEBA/chloroform), (DGEDDS/chloroform) and (DGEDDP/chloroform) all exhibit the same rheological properties regardless of the temperature of (polymer/solvent) system. Whereas for (TGMDA/chloroform), (TGEDA/chloroform) and (TGABSA/chloroform) systems the viscosity increases with temperature. This is due to the development of the homopolymerization reaction, since the viscosimetric behavior elevates as the molecular weight of the solute increases. Bekhta et al. [20] have shown that the viscosimetric properties also increase with the weight percentage of the trifunctional epoxy resin, triglycidyl trimercapto-ethanol ether of phosphorus (TGETMEP) (Figure 3) incorporated in the system (TGETMEP/methanol). TGETMEP matrix was synthesized and identified by Bekhta et al. [20]. Other researchers have studied viscosimetric behaviors as a function of mass percent and as a function of temperature. The viscosity properties increase by increasing of the percentage of polymer in the (polymer/solvent) systems [21, 22]. Also, other authors investigated the shear rate and temperature-dependent dynamic viscosity and shear stress properties of two mono and difunctional epoxy polymers such as novolac epoxy polymer (NEP) and diglycidyl ether of bisphenol A (Figure 4), respectively [23, 24]. These results showed that the increase in shear rate and temperature leads to a reduction in dynamic viscosity and strain stress for both molecular matrices. Also, dynamic viscosity and shear stress of polymers decrease with increasing shear rate and temperature. Then, they also determined the macroscopic mechanical properties by studying the micro and nano structure of the material. Hsissou et al. [25] showed the viscoelastic property of the phosphorus prepolymer namely pentaglycidyl ether pentaphenoxy of phosphorus (PGEPPP). PGEPPP has been synthesized and characterized according the procedure reported in literature (Figure 5) [25]. They have shown that the viscoelastic behavior decreases with increasing temperature, these results show a three phase pattern: (vitreous state, transition state and that of flow). This can be explained by the heat released by the device. These results are in good agreement with data obtained by Bekhta et al. [26].

## 3 Rheological properties

### 3.1 Rheological behavior of composite reinforced by organic filler (HGCP)

**Figure 6:**
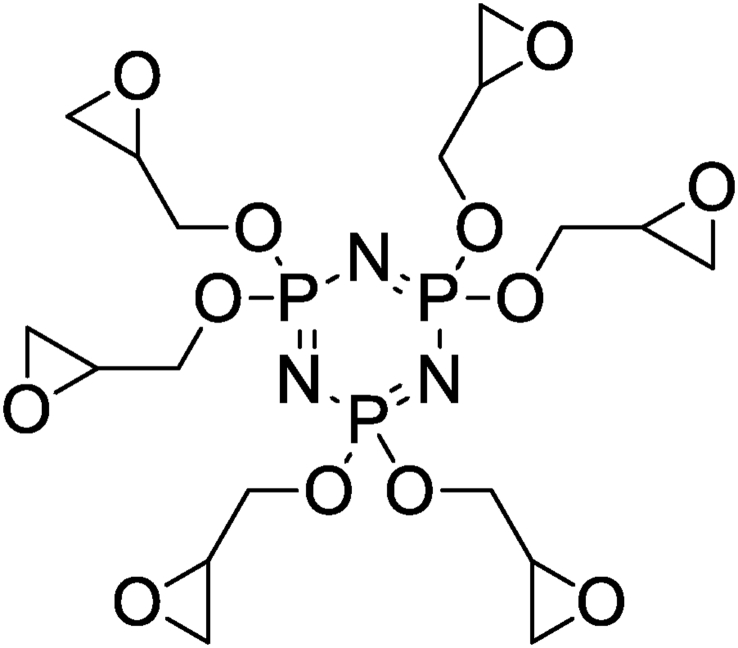
Structure of HGCP [27].

El Gouri et al. [27] elaborated and formulated the composites based on the DGEBA as prepolymer, hexaglycidyl cyclotriphosphazene (HGCP) as organic filler and the methylene dianiline as hardener. Hexaglycidyl cyclotriphosphazene has been synthesized and characterized according the procedure reported in literature (Figure 6) [27]. These results were obtained by employing a sufficiently low constant shear stress of 0.1% so as not to affect the properties of the composite material in the linear viscoelastic area. The storage modulus and loss modulus of composite polymers increase with increasing in frequency. The results showed that all mixtures of 0%, 5%, 10% and 15% HGCP behave like a solid, with the rheological behavior (G′ and G″) almost independent of angular velocity. The occurrence of G′ and G″ has been determined by several authors [28, 29, 30].

### 3.2 Rheological behavior of nanocomposite reinforced by carbon nanotubes (CNT)

Grich et al. [31] showed the rheological property of nanocomposites consisting of a dispersion of carbon nanotubes (CNT) particles in the difunctional epoxy polymer DGEBA. These rheological behaviors have determined the structures and relationships responsible for CNT curing of polyepoxide prepolymers [32, 33, 34]. This method is the key to know and the development of the implementation of nanocomposites. G′ and G″ elevate with varying formulations of the NTC incorporated in the DGEBA matrix. The rheological properties of hybrid nanocomposites increased linearly with the percentage of the charge used. These data are in good agreement with the data obtained by varying authors for carbon nanotube charge dispersion [35, 36]. The authors observed microscopic aggregated structures of CNT in a shear flow for dispersion of untreated CNT [37]. On the other hand, they observed a uniform dispersion of treated CNTs. The properties of dispersed CNT systems have been showed by different authors. The authors studied that the rheological behaviors depend with technique of dispersion of CNTs in polymer matrix [38].

### 3.3 Rheological behavior of nanocomposite reinforced by PTS, TiO_2_ and NP

**Figure 7:**
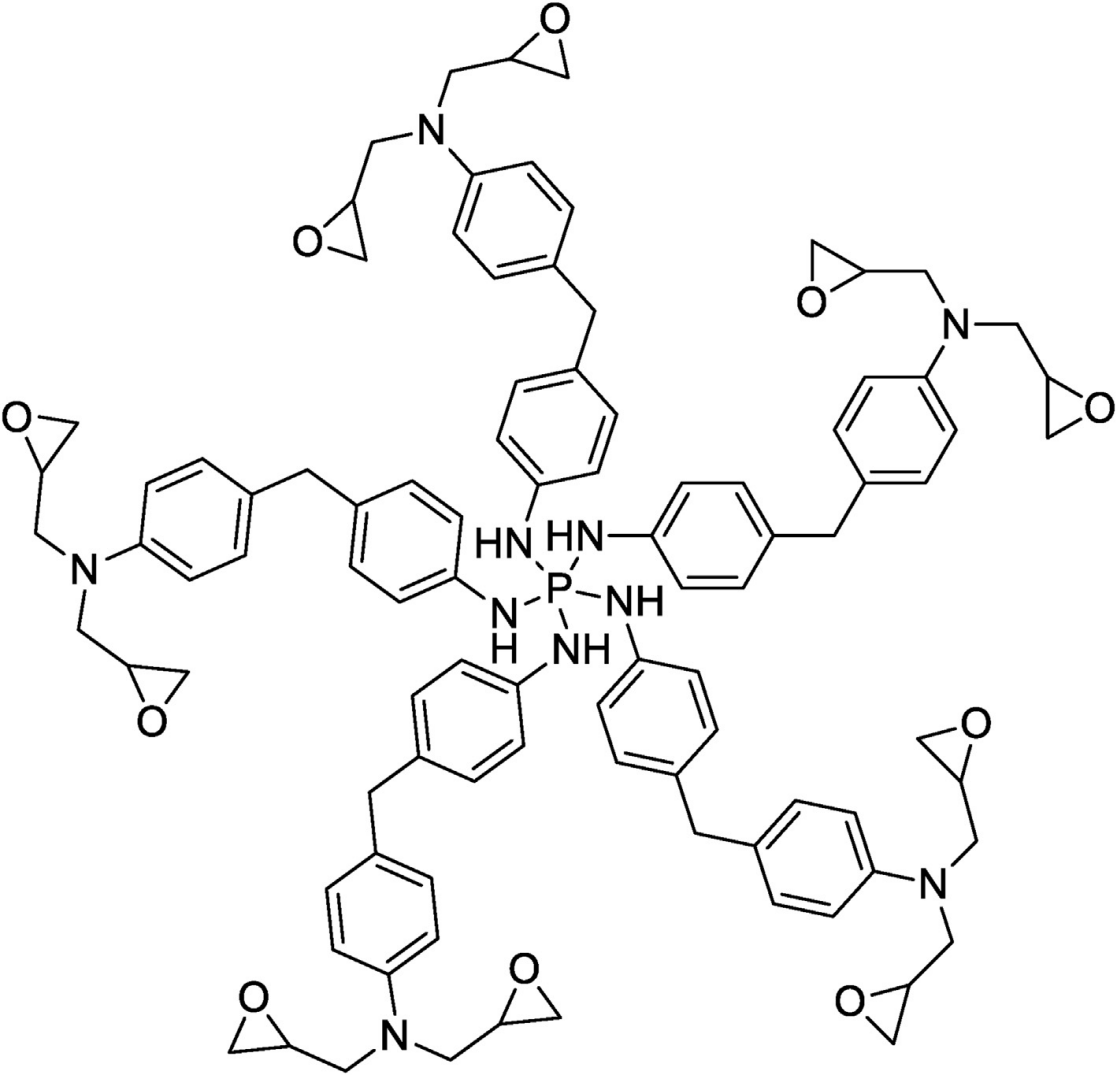
Structure of DGPMDAP [39].

**Figure 8:**
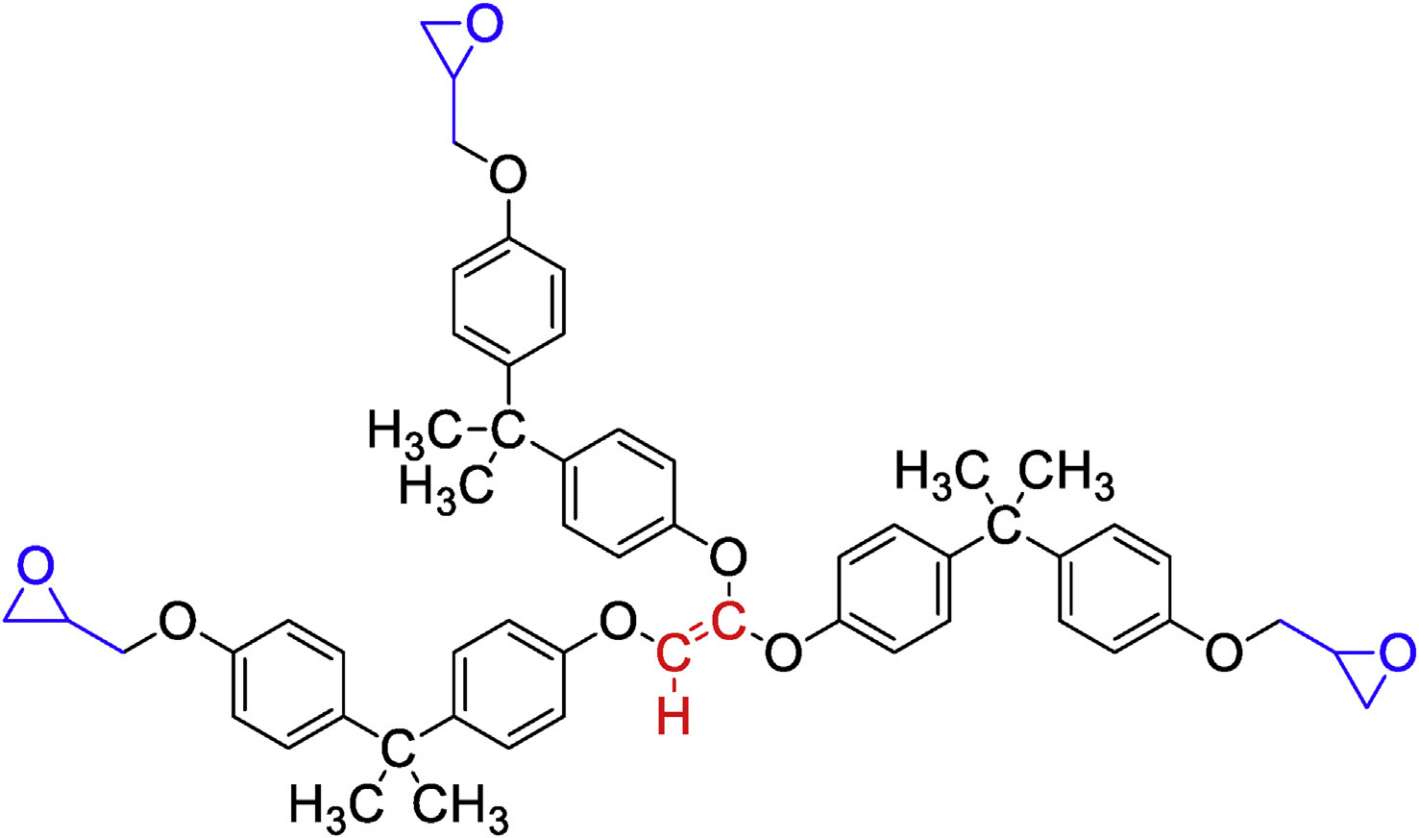
Structure of TGEEBA [40].

**Figure 9:**
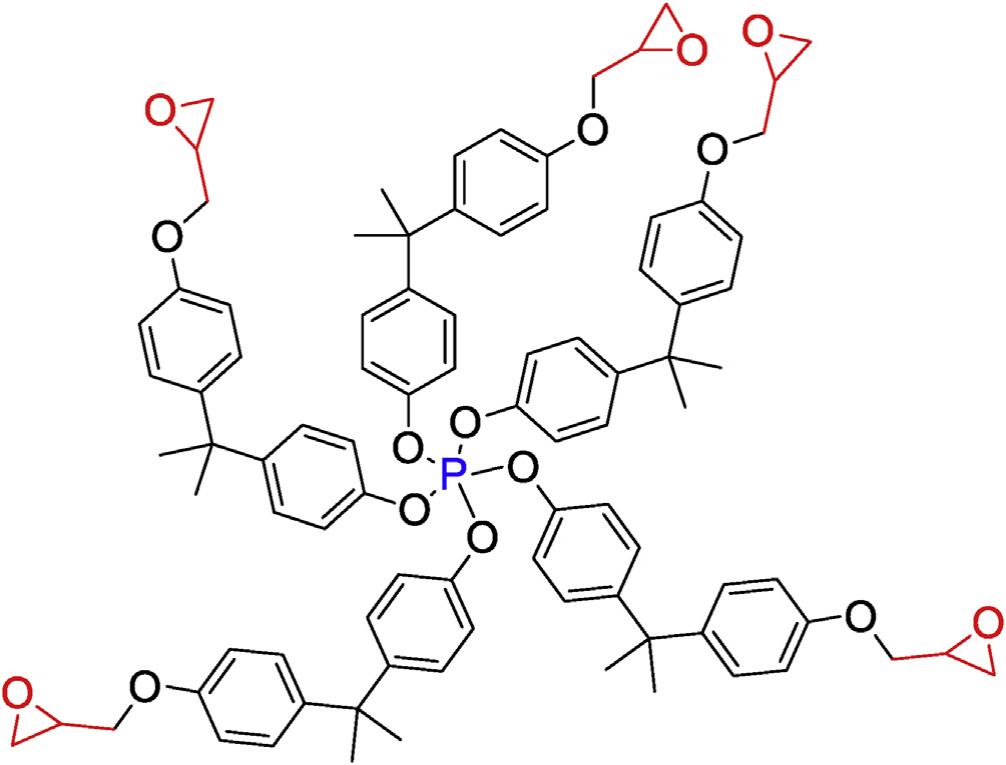
Structure of PGEPBAP [41].

**Figure 10:**
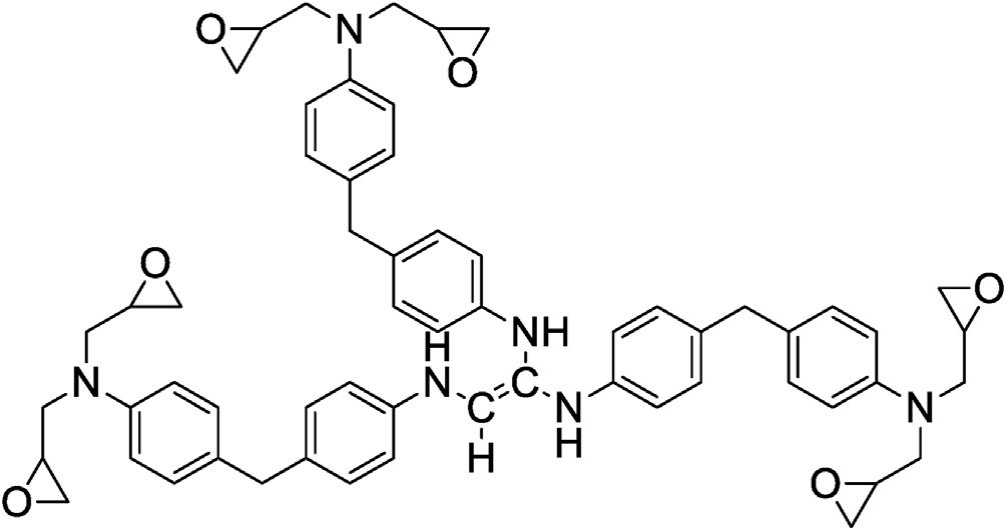
Structure of HGEMDA [42].

**Figure 11:**
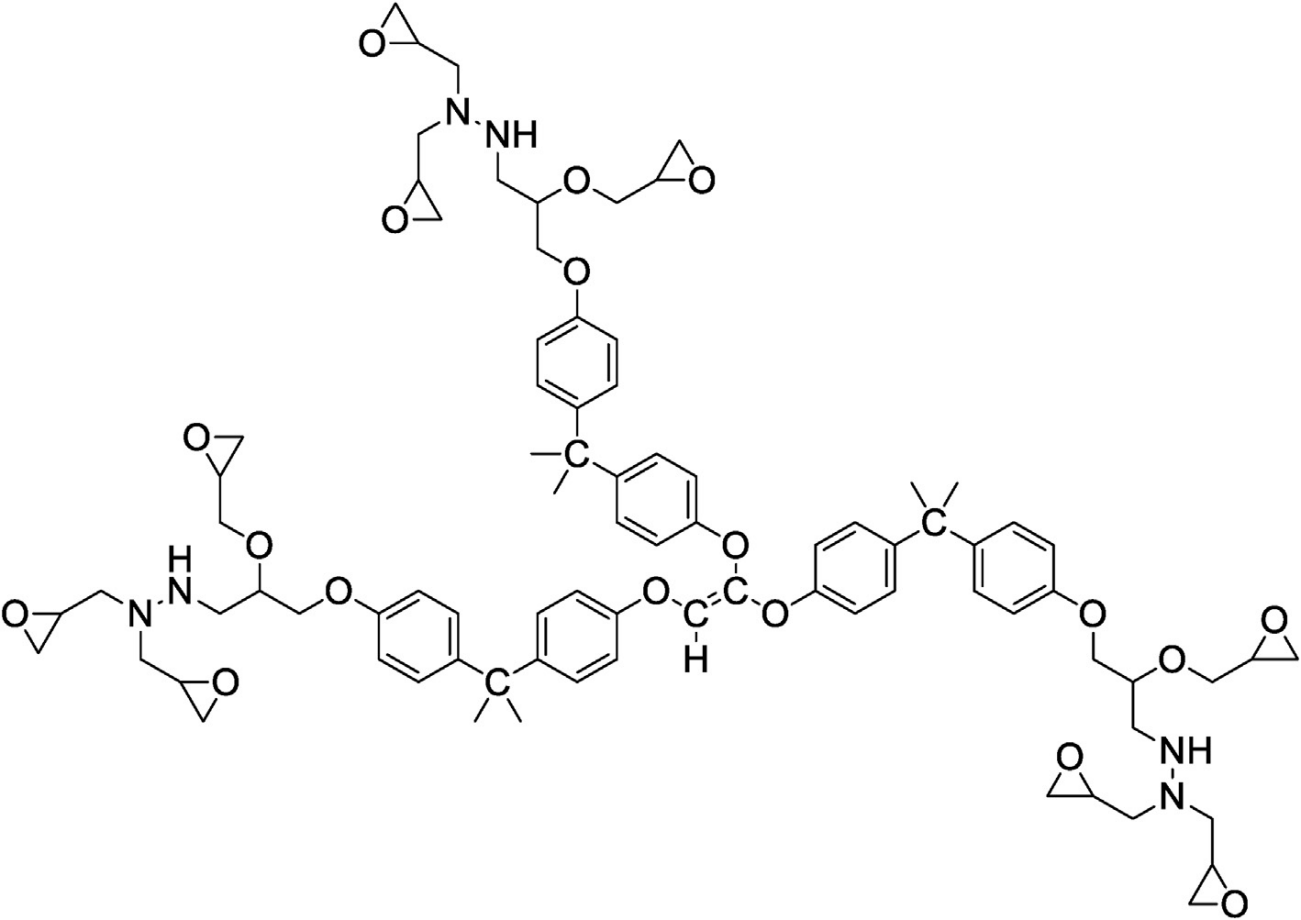
Structure of NGTHTPTBAE [43].

Bekhta et al. and Hsissou et al. [20, 39] studied the rheological properties according to angular velocity of the two prepared nanocomposites (TGETMEP/MDA/PTS) and (DGPMDAP/MDA/TiO_2_) based on triglycidyl ether trimercapto ethanol phosphorus (TGETMEP) and decaglycidyl pentamethylene dianiline of phosphorus (DGPMDAP) (Figures 4 and 7), respectively. TGETMEP and DGPMDAP matrices were synthesized and identified by Bekhta et al. and Hsissou et al. [20, 39]. These resins are curing using 4,4-diaminodiphenyl benzene (MDA) as a curing and formulated by two fillers (trisodium phosphate (PTS) and titanium dioxide (TiO_2_)) at varying percentages. The results showed that the G′ and G″ elevate with the addition of inorganic charges in prepared nanocomposites (TGETMEP/MDA/PTS) and (DGPMDAP/MDA/TiO_2_). Further, other authors investigated the G′ and G″ of different (TGEEBA/MDA/PN), (PGEPBAP/MDA/PN), (HGEMDA/MDA/PN) and (NGTHTPTBAE/MDA/PN) hybrid composites derived from tri, penta, hexa and nanofunctional such as TGEEBA, PGEPBAP, HGEMDA and NGTHTPTBAE (Figures 8, 9, 10, and 11), respectively [40, 41, 42, 43]. These latter are crosslinked by 4,4-diamino diphenyl benzene and formulated by PN. The results showed that the addition of natural phosphate as filler in (TGEEBA/MDA/PN), (PGEPBAP/MDA/PN), (HGEMDA/MDA/PN) and (NGTHTPTBAE/MDA/PN) elaborated composites give interesting rheological properties. Various authors demonstrated that the inorganic charge incorporated in the composites elevates the G′ and G'' [39]. Then, viscosity properties of three matrices diminish with elevating temperature. Then, the viscoelasticity elevates with the number of the constituent epoxy group in tri, penta, hexa and nano epoxy polymers with three, six and nine functional groups, respectively. The rheological behavior of hybrid nanocomposite polymers depends on fiber content, fiber length, fiber orientation, fiber-to-matrix bonding, fiber configuration and filler.

### 3.4 Rheological behavior of nanocomposite reinforced by pulp fiber suspensions

Mohataschemi et al. [44] showed the rheological property of two types of nanocellulose, crushed Masuko and oxidized TEMPO, respectively. The results obtained are in agreement with experiments. The author showed that composites exhibit gel-like properties over a wide range of frequencies [45]. Also, other authors studied the G′ and G″ of pulp fiber suspensions. These suspensions are important complex fluids in the manufacture of several pulp fibers. These properties are the keys to fiber suspensions such as behavior regimes based on inter fiber, yield strength, viscosimetric behaviors [46, 47, 48].

### 3.5 Rheological behavior of nanocomposite reinforced by multiwall nanotube

Lim et al. [49] studied the nonlinear response of nanocomposite polymers under high amplitude oscillatory shear flux. They used different analytical methods to study poly (caprolactone/multiwall nanotubes) nanocomposites (PCL/NTMP) under (COGA) flux. They calculated the nonlinear factor from the rheology of the Fourier transform according to deformation amplitude and zero deformation nonlinearity, respectively. They compared linear and nonlinear viscoelastic properties by increasing the concentration of the multiwall nanotube. They studied the effect of NTMP on the nonlinear viscoelastic behaviors of composite polymers such as (PCL/NTMP), (PCL/MOM) and (PCL/PCC), respectively.

### 3.6 Rheological properties of colloidal and noncolloidal suspensions

Jamali et al. [50] studied the rheological behavior of colloidal by computer model. They showed that for suspensions of hard quasi-spheres, the colloidal pressure and shear stress tends towards close to the maximum fraction. Ovarlez et al. [51] studied the rheological property of noncolloidal spheres in boundary fluids. They studied stress suspension flows in a concentrated cylinder Duvet geometry using magnetic resonance imaging techniques. Other search proposed a theoretical approach for isotropic suspensions, to describe suspensions in simple shear flows [52]. They observed the accordance between experimental data and theoretical approache. They also showed that the addition of particles induces new emerging characteristics in the rheology of the yield stress fluid.

### 3.7 Rheological behavior of nanoparticles and PAL

Srivastava et al. [53] showed the rheological behavior of nanoparticles attached to oligomers suspended in a low molecular weight polymer host at varying particle sizes and particle loads. The small size of nanoparticles cores is considerable for know the step durability and rheological behavior. Cailloux et al. [54] demonstrated the dynamic viscosity of the polylactic acid prepared by reactive extrusion. They measured the rheology of non-uniform branched macromolecules, containing relatively short-chain branching, for confirmation that conventional chromatographic and spectroscopic techniques had limited sensitivity. They used the thermorheological behaviors which are globally unchanged for PAL-REX. They observed the wider transition zone of complex viscosity function in all PAL-REX samples. They proposed the Yassuda-Tile model which is an extension of the Havriliak-Negami model, which were realized to catch the properties of complex viscosity. Nouri et al. [55] focused on improving the rheology of the polylactic acid (PAL) melting process using two strategies. They showed that the addition poly-L-lactic acid (PLAL) increases the shear thinning, shear viscosity and elasticity pattern of linear PLAL at the same time. The authors have synthesized poly-D-lactic acid (PDAL) in presence of analogous macromolecular polymers. Indeed, the presence of branched architectures, physical cures owing to the development of stereo-complexes betwixt PLAL and PDAL matrices. Finally, they observed that the improvement of the rheological properties of polylactic acid is more important for mixtures containing a stereo-complex structure.

### 3.8 Rheological behaviors of micellar solutions

Zhao et al. [56] characterized the linear and nonlinear rheological behaviors of cetyl trimethyl ammonium bromide cationic surfactant and 3-hydroxy naphthalene-2-carboxylate (HNC) organic salt. They showed that the naphthalenic and high hydrophobicity incorporated in CNT induce considerable development of cetyl trimethyl ammonium bromide and promote the formation of a stable micellar network [57, 58, 59]. Gaudino et al. [60] studied the rheology of micellar systems composed of sodium salicylate and cetylpyridinium chloride (SalNa-ClCP) in aqueous solutions by changing the concentrations of the salts. They determined zero shear viscosity as a viscoelastic response as a function of SalNa concentration, they also showed that the Newtonian viscosity depends on the salt concentration (SalNa-ClCP). They showed that the elastic plateau model remains essentially constant for a strongly decreasing relaxation of the system, the rate of micelle breakage decreases. Yesilata et al. [61] described the nonlinear shear and extensive flow dynamics of a micellar solution based on erucyl bis (2-hydroxy ethyl methyl ammonium) chloride. They used the low amplitude oscillatory shear experiment for the determination of linear viscoelastic parameters. They showed that the shear rheology is stable and time dependent, the measurements of extensional transient flow are performed in an extensional rheometer with capillary rupture. Micellar fluid samples show strong hysteresis behavior in increasing and decreasing shear stress. They measured the instantaneous shear velocity for different values of shear stress, evolving surfactant solutions in the geometry of the flow structure. Finally, they studied the dynamics of capillary rupture of micellar liquid samples in an extensional flow [62].

### 3.9 Linear and nonlinear viscoelastic of nanocomposites reinforced by clay and silica

Salehiyan et al. [63] studied the linear and nonlinear viscoelastic behaviors of polypropylene/polystyrene blends using dynamic oscillatory shear flow. They used both different nanoparticles (organomodified clay and fumed silica) at different concentrations. They analyzed the nonlinear stress under high amplitude oscillatory shear flux (COGA), the nonlinearity was calculated from Fourier transform rheology. They used the nonlinear linear viscoelastic ratio (normalized nonlinear viscoelasticity/normalized linear viscoelasticity) to quantify the degree of dispersion of different particles at different concentrations in nanocomposites. The authors determined the relationship between the value of the linear nonlinear viscoelastic ratio and the size of the PS droplets in the PP matrix [64]. The author studied linear and nonlinear rheological properties in the presence of silica nanoparticles of different natures (hydrophilic and hydrophobic) on mixtures (PP/PS) [65]. They obtained the two linear and nonlinear rheological properties of the tests small amplitude oscillatory shear (COPA) and large amplitude oscillatory shear (COGA), respectively. They determined that the values of the linear nonlinear viscoelastic ratio are equal to 1 for an increasing concentration of hydrophilic silica (OX50) in the mixtures (PP/PS/OX50) without altering the droplet size and much greater than 1 by increasing the concentrations of hydrophobic silica nanoparticles (D17 and R202) in the mixtures (PP/PS/D17 + R202) which is consistent with the evolution of the morphology, respectively.

Park et al. [66] have recently studied the rheology of multi block olefin copolymers. They used data for the determination of the effect of octene monomer content on rheological behavior and the occurrence of the mesophase separation transition in the melt. To distinguish between crystallization and mesophase separation transition, they measured rheological properties over a temperature range. These results confirmed the rheological properties of the mesophase separation transition present in the melt at temperatures well above the melting point. Sim et al. [67] studied the behavior of large amplitude oscillatory shear (COGA) of electrorheological (ER) fluids using a three dimensional dynamic simulation of the particles. They used a dynamic mode with large deformation, to understand the behavior of high amplitude oscillatory shear and its mechanism for the development of an efficient electrorheological fluid. Other author applied a high amplitude oscillatory shear stress for solutions of anionically synthesized linear polystyrene in dioctyl phthalates [68].

### 3.10 Rheological behavior of dilute and semi-dilute dispersion of colloids

Larson [69] studied the rheological behavior of flexible polymers, bead and bead models presented the scope of applicability and inclusion methods for hydrodynamic interactions. After reviewing and updating the work in the linear viscoelastic regime, the main focus shifts to the more complex nonlinear regime. They concluded that the hydrodynamic and volume interactions excluded in polystyrene coils are more flexible and therefore more condensed. Varga and Swan [70] studied the rheological properties of colloids with short range attractions. They calculated the complex viscosity of the materials by studying their response to low oscillating shear. They used first-order expansion at low strain rates to resolve the microstructure and stress in the dispersion. They exhibited a viscoelastic response when moving from weak to strong attraction, the increase in inter-particle attraction reduces viscosity at low frequencies.

### 3.11 Rheological behavior of sheared granular flows

De Cagny et al. [71] studied the granular as a function of magnetic resonance imaging for non-locality. Measurements showed that the local volume fraction of the systems becomes heterogeneous throughout flow. They concluded that dry granular system is low strong; however it become visible that apparent non local behaviors are simply owing to development of a shear bond result in granular expansion [72]. Other author provided an image compatible with the local rheology at high shear rates; operated in vicinity of interference, where the representation of local rheological is short [73, 74]. Sarkar et al. [75] have successfully described the model of soft vitreous rheology, the time as a function of shear rheological of complex fluids. So, the vitreous rheology of fluid is widely exponential division of trap energy and the rheological of organic nano particulate hybrid materials is best described by a narrower energy distribution.

## 4 Conclusion

After analyzing a large number of literatures on the viscosimetric and rheological behaviors on polymers and hybrid nanocomposite polymers, we have concluded the following points:

✓ The viscosity properties increase by increasing of the percentage of polymer in the (polymer/solvent) systems.
✓ The dynamic viscosity and shear stress of polymers decrease with increasing velocity gradient and temperature.
✓ The storage modulus and loss modulus of polymers elevate with increasing in frequency.
✓ The rheological properties of hybrid nanocomposites increased linearly with the percentage of the charge used.
✓ The rheological behavior of hybrid nanocomposite polymers depends on fiber content, fiber length, fiber orientation, fiber-to-matrix bonding, fiber configuration and filler.
✓ The application of hybrid nanocomposite polymers as an alternative nanocomposite material, especially in flow and processing phenomena is very reasonable with the high strength, low cost and environmentally friendly production.

## Declarations

### Author contribution statement

All authors listed have significantly contributed to the development and the writing of this article.

### Funding statement

This research did not receive any specific grant from funding agencies in the public, commercial, or not-for-profit sectors.

### Competing interest statement

The authors declare no conflict of interest.

### Additional information

No additional information is available for this paper.
